# A Reappraisal of How to Build Modular, Reusable Models of Biological Systems

**DOI:** 10.1371/journal.pcbi.1003849

**Published:** 2014-10-02

**Authors:** Maxwell L. Neal, Michael T. Cooling, Lucian P. Smith, Christopher T. Thompson, Herbert M. Sauro, Brian E. Carlson, Daniel L. Cook, John H. Gennari

**Affiliations:** 1 Department of Bioengineering, University of Washington, Seattle, Washington, United States of America; 2 Auckland Bioengineering Institute, University of Auckland, Auckland, New Zealand; 3 Department of Physiology, Medical College of Wisconsin, Milwaukee, Wisconsin, United States of America; 4 Department of Molecular and Integrative Physiology, University of Michigan, Ann Arbor, Michigan, United States of America; 5 Department of Physiology and Biophysics, University of Washington, Seattle, Washington, United States of America; 6 Department of Biomedical Informatics and Medical Education, University of Washington, Seattle, Washington, United States of America; National Cancer Institute, United States of America and Tel Aviv University, Israel

## Overview

Biological researchers increasingly rely on computational models to integrate biological systems knowledge, test hypotheses, and forecast system behavior. The expanding size of these models requires solutions for managing their complexity. Modularity, a time-tested design principle for managing complexity, can be applied within the biological modeling field to parallelize work, automate composition, and promote effective model sharing. As modelers of complex biological systems, we aim to apply modular production to accelerate our efforts and have therefore investigated several currently available approaches for modular modeling. We argue that some traditional features of modularity, in particular the isolation of a module's contents from the rest of the system, can impede model sharing and composition when applied within the context of biological simulation. Alternative approaches that can automatically interface model components based on the biological meaning of their contents (their semantics) avoid these limitations. Our conclusions have strategic implications for the design of systems biology, synthetic biology, and integrated physiological modeling technologies, as well as community-level model curation efforts.

## Introduction

Given the enormous increase in systems-level biological information in recent years, along with concurrent advances in computing power, researchers can now simulate biological systems on an unprecedented scale. Contemporary models may contain hundreds or thousands of variables and equations, and as these simulations grow in scope and fidelity, managing model complexity becomes increasingly difficult. A solution to this challenge is to apply modular modeling approaches that break up complex models into more manageable pieces, parallelize work, and enable more automated model composition. As a design principle, modularity is a powerful and time-tested approach for managing complexity. For example, the aircraft, automobile, and electronics industries all rely on modular product development; mass production of their complex goods is impossible without it. We, as modelers of complex biological systems and as members of larger research communities developing systems biology, synthetic biology, and multiscale physiology models, are interested in applying this same production philosophy to facilitate model reuse among the greater modeling community and increase research productivity. Modular modeling offers an opportunity to move beyond traditional modeling practices characterized by hand-coded, custom-made models with limited capabilities for reuse. Our vision is to utilize the expanding set of publicly available biological models as a library of interoperable modeling components that can be easily recombined to build complex, integrated simulations of biological systems.

Working towards this vision, we have explored several technologies for modular modeling to better understand how we might implement a general approach for automated model composition and community-level model sharing. Based on our exploration, we assert that information-hiding interfaces, one of the hallmarks of traditional modular designs because of the advantages it confers, can actually impede reuse of biological models. In a biological context, such interfaces are problematic because they can hide critical points of coupling between modules in the same system. Working from this argument, we describe an alternative approach that can address this problem.

## Modularity and Interfaces

Modularity, despite being an intuitive design principle, can be difficult to formally define. Here we summarize and apply a definition articulated by Baldwin and Clark [1] that was adapted from McClelland and Rumelhart [2]. According to this definition, there are two central ideas that comprise the concept of a module:

1. A module is a unit in a larger system that retains an individual identity but interacts with other system units.
2. The complexity within a module is isolated from the larger system but made accessible via an interface; the interface defines how the unit can interact with the larger system.

Modeling components that adhere to this definition are suitable, and perhaps optimal, when researchers agree on what the appropriate coupling points and interfaces should be for larger model compositions. However, we argue that for broader reuse of models across research groups, or within groups that have evolving modeling needs, these types of modules can be problematic. Our reasoning is as follows:

By the above definition, a module's interface defines how it interacts within a larger system. This presupposes two things. First, that the “larger system” has already been defined. However, because there is no way to anticipate all the ways that the biological modeling community may repurpose a publicly available modeling component, there is no way to define the system in which that component participates a priori. Second, that there is a universal and unchanging agreement about what constitutes a module and how such modules communicate via predefined interfaces. Our concern is that current computational implementations of modules and interfaces, while suitable for specific purposes, prematurely commit modelers to modeling components that may be difficult to recombine and repurpose in the face of changing knowledge and evolving modeling needs. For example, biological information from a new experiment may raise the importance of a variable hidden within a component. Therefore, it becomes difficult to anticipate what information in a component should be exposed or hidden by an interface.

**Figure 1:**
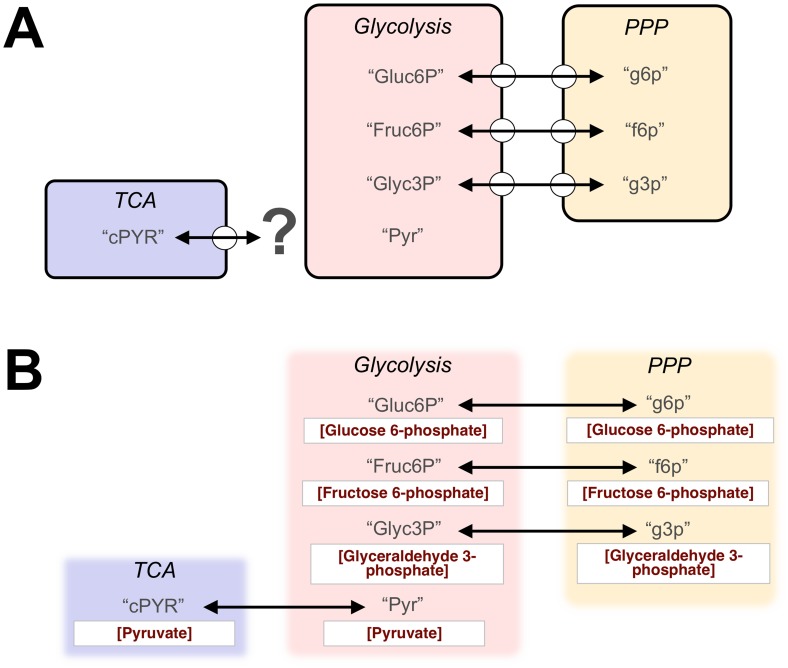
A modular model composition task using traditional, information-hiding approaches versus semantics-based, adaptable interface modularity (SAIM). A: Predefined interfaces applied to publicly-available glycolysis, pentose phosphate pathway (PPP) and tricarboxylic acid cycle (TCA) modules may allow appropriate computational linkage (double-headed arrows) between the first two models but prevent linkage with the third. The interfaces on the glycolysis and PPP models expose codewords representing the concentrations of glucose 6-phosphate (“Gluc6P,” “g6p”), fructose 6-phosphate (“Fruc6P,” “f6p”), and glyceraldehyde 3-phosphate (“Glyc3P,” “g3p”) but conceal the glycolysis model codeword representing pyruvate concentration (“Pyr”), a critical coupling point between the glycolysis and TCA cycle models. B: Using SAIM, all modeling elements are exposed and semantically defined, allowing biologically consistent coupling of the three modeling components at the time of composition.

For example, researchers studying nucleotide synthesis might repurpose a glycolysis model for integration with a pentose phosphate pathway (PPP) model, while another research group investigating metabolism might integrate it with a tricarboxylic acid (TCA) cycle model. Both are valid modeling tasks, and modeling approaches for community-level reuse should be able to accommodate both. If the interface to the glycolysis model is specified ahead of time, this can limit its reuse. Let us assume the model was initially intended to link with a PPP module and includes an interface that exposes three variables: the concentrations of glucose 6-phosphate, fructose 6-phosphate, and glyceraldehyde 3-phosphate. Such an interface hides a critical point of coupling between the glycolysis and TCA models: there is no way to integrate them so that the TCA cycle's input pyruvate concentration becomes dependent on the glycolysis model's dynamics (Figure 1A). The interface on the glycolysis model, although quite suitable for the original modeling task, hides biological knowledge critical to its reuse for modeling tasks beyond its initial design goals. Besides hiding the pyruvate concentration variable “Pyr” in Figure 1, the interface may hide other variables, such as ATP and ADP concentration, which can be critical for connecting the glycolysis model to others in a biologically consistent fashion.

The use of such predefined interfaces is a standard approach in component-based software engineering. This is known as “encapsulation” and allows software developers to work independently on a system's components without requiring them to understand the details of other components or accommodate changes to the internals of components developed externally. We do not argue against such predefined interfaces, per se, as they can be useful for composing models, organizing numerical solutions, and simplifying communication about biological systems among researchers. We also acknowledge that in forward-engineering tasks, in which engineers link modules with predefined interfaces to create novel composite systems (such as modeling a novel electronic circuit), it is not necessary to specify all potential composite systems ahead of time for the interfaces to be useful. Nonetheless, these interfaces limit the number and type of compositions available to the engineer; they prevent editing or expanding the mathematical machinery hidden within the modules and can prevent effective interfacing between modules coded for different compositional packages. Very often, these are exactly the modeling tasks that biological modelers face when attempting to reuse shared models. For example, a modeler may wish to increase a model's biological detail to improve its predictive accuracy or integrate multiple, related models coded by different research groups to explore a system more holistically.

A solution to the problems introduced by predefined interfaces is to “white-box” module contents and to label the contents with their biological meanings—their semantics. We call this new approach “semantics-based, adaptable interface modularity,” or SAIM (Figure 1B). As we illustrate in the following sections, SAIM can accelerate and facilitate the repurposing of legacy and emerging models by representing their biological content free of imposed computational interfaces. SAIM leverages these semantic descriptions to propose “on-the-fly” interfaces between merged components that are specific for particular, even transitory, modeling purposes. The advantage we foresee is a major increase in the ease and speed by which modelers across the modeling community can reuse available modeling code.

In the following section we discuss our exploration of modular biological modeling technologies so as to place SAIM within the context of the broader effort to build modular biological systems from reusable models.

## Current Modular Modeling Technologies

In our exploration of modular modeling technologies, we focused on those that use declarative model description formats. These formats, in contrast to imperative (procedural) formats, describe models in a more sharable fashion, allow for model exchange across modeling software, and separate the model description from analysis and visualization workflows. These characteristics are important for sharing models because the broader biological modeling community uses a variety of modeling software tools and computational research pipelines.

Among the modular modeling technologies we explored, we found three distinct types of modular composition, which we discuss in turn.

### Type 1: Black-box, code-level coupling using information-hiding interfaces

In these types of compositions, information-hiding interfaces are applied to modeling components and the modeler links the components according to model variables' input/output assignments. This is the standard approach for component-based software engineering and is often used by modelers working in procedural, object-oriented languages such as MATLAB, Java, C++, etc. It is also a compositional approach supported by CellML [3], a declarative, XML-based modeling format developed for the International Union of Physiological Sciences (IUPS) Physiome effort [4] that includes explicit constructs for delineating reusable modeling components with predefined interfaces. CellML modeling tools such as OpenCell [5] (http://www.opencell.org) and Cellular Open Resource (COR) [6] (http://cor.physiol.ox.ac.uk/) use these interfaces to guide and validate the model composition process.

The unit of modular composition in CellML models is the “component” element. Each CellML model must declare at least one component, and all model variables and equations are declared within components. Each component is a computational block within the model with a predefined interface that indicates whether a variable is an input, output, or hidden. A CellML component may represent a distinct computational or biological aspect within a model. As of CellML version 1.1, modelers can reuse any component in a CellML model through the CellML “import” element. This creates a new instance of the component in the model, linking in the component's computational machinery. Components can also be arranged hierarchically using, for example, an encapsulation relationship.

Importing components, as performed in OpenCell, reduces modeling costs by allowing modelers to refer to previously developed model components rather than code them anew. For an example of this kind of composition, see Cooling et al. [7]. When a component is imported into a new model, the modeler can then specify connections that map the imported component's exposed variables to other exposed variables in the model. This links the imported component to the rest of the model's mathematical structure.

### Type 2: White-box, code-level coupling

In this type of model composition, information-hiding interfaces are absent and all code-level elements of a modeling component are available as potential coupling points with other components. As in Type 1 compositions, the modeler establishes links between models by creating mappings between code-level constructs such as model variables. Technologies that support this type of composition include Antimony [8] (http://antimony.sourceforge.net), the Systems Biology Markup Language (SBML) Hierarchical Model Composition package (SBML-comp—http://sbml.org/Documents/Specifications/SBML_Level_3/Packages/comp), and TinkerCell [9] (http://www.tinkercell.com).

Part of the reason Antimony and SBML-comp apply a white-box approach is to support compositions of SBML models. SBML, which is an XML-based modeling format designed to facilitate reuse of molecular systems models across software tools, intentionally excludes constructs for information-hiding interfaces. One reason for this is that SBML has long supported the reuse of models in new contexts and is intended to allow different computational implementations of the same model. For example, an SBML model can be solved in a continuous or a stochastic manner, depending on the modeler's needs. Core SBML specifies the reactions in the model, their participating chemical species, their kinetic rates, and any necessary kinetic parameters. It leaves the model's full computational implementation, including the construction of the necessary conservation equations, to external simulation software tools. SBML's exclusion of delineated model components and interfaces also allows for maximal flexibility and customization when interconnecting SBML models or decomposing them into reusable subcomponents [10]. (This is also the rationale behind TinkerCell's white-box approach for supporting compositions of synthetic biology models.) However, white boxes require modelers to take explicit care that new connections do not violate assumptions inherent in the models being connected. For example, some models are only accurate if the biological outputs stay within a certain range. If a model is connected to another that causes the first to exceed that range of accuracy, the combined model may be biologically invalid. If Type 1 (black-box) composition is desired, Antimony, SBML-comp, and TinkerCell all provide the ability to define a suggested interface, to which the modeler may choose to restrict themselves. But in all three systems, nothing prevents a modeler from creating new mappings to otherwise internal elements.

The Antimony software package [8] allows modelers to create hierarchical model compositions from SBML as well as CellML source models. To build composite systems, modelers use Antimony's shorthand text commands to manually identify and declare links between code-level elements in component models. Antimony provides a nonbinding, suggested interface for hierarchical models, allowing modelers to choose whether to use Type 1 or Type 2 compositions, or some combination of the two. It does not impose any particular interface on source SBML models and it programmatically exposes the contents of CellML's black boxes—converting them into white boxes—so that users may couple these models as their research tasks demand. Importantly, this process requires users to have a detailed, code-level understanding of each model's contents before interconnecting them into a larger system. These same contingencies apply to SBML-comp and TinkerCell.

### Type 3: White-box, biological-level coupling

As in Type 2 compositions, the computational elements of the modules used in Type 3 compositions are all exposed (white-boxed) as potential coupling points. What distinguishes these modules from purely white-box, Type 2 modules, is that machine-readable annotations are applied to each model that help guide the creation of biologically consistent links between modeling components. This is designed to expedite the composition process by automatically proposing a set of couplings between modeling components and by allowing modelers to work at the biological, rather than computational, level of abstraction. In other words, modelers are not required to know the idiosyncratic names of model elements (variables, etc.) to link systems together. Instead, they can perform their compositions based solely on what is represented by the model, biologically (Figure 1B).

The semanticSBML toolkit [11] is one technology that supports this type of composition (http://www.semanticsbml.org). Curated SBML models in the BioModels database [12] are rich in machine-readable, semantic annotations that define their species, reactions, etc. When merging models with semanticSBML, the software automatically identifies biologically equivalent reactions and chemical species among the models to be merged. The user then chooses which mathematical representation of these commonalities they wish to preserve in the merged system (otherwise, the merged model would be biologically overdetermined). This process establishes the links that couple the models. By establishing model–model connections at the semantic level, semanticSBML reduces the need for modelers to develop code-level knowledge of modeling components.

The semanticSBML toolkit was designed to work with SBML models; thus, its focus is on composition of chemical network models. SemGen [13]–[15], a software suite for semantics-based model annotation, composition and decomposition, is intended to provide these capabilities for a broader range of models and modeling languages (http://sbp.bhi.washington.edu/projects/semgen). With SemGen, users can perform Type 3 compositions with models that adhere to the SemSim framework [13]–[15], a semantics-based knowledge architecture designed to explicitly capture the computational and biological aspects of simulation models across biological scales. Currently, SemSim models are represented and stored in the Web Ontology Language (OWL [16]), but could theoretically be encoded in other knowledge-representation formats such as the Open Biomedical Ontology format (http://www.geneontology.org/GO.format.obo-1_2.shtml) or Turtle (http://www.w3.org/TR/turtle/). In the SemSim approach, annotations against biomedical ontologies are created to compose precise, descriptive definitions of the biophysical phenomena simulated in a model. As with semanticSBML, when SemSim models are integrated into a merged system, these annotations are used to couple the models in a biologically consistent manner. SemSim model variables are not assigned an explicit input/output status a priori, nor are they hidden by interfaces. Instead, the interface between merged models is created at the time of composition and depends on the biological commonalities they share.

The merging task presented in Figure 1B provides a specific example of this type of composition. By examining the annotations on the model variables, a Type 3 composition tool could identify where the models overlap, biologically. For example, the TCA and glycolysis models both simulate pyruvate concentration. With these biological commonalities identified, the user then chooses which mathematical representation of the concentration they wish to preserve in the merged system. Let us suppose that pyruvate concentration is a static constant in the TCA model but a dependent variable in the glycolysis model. If the user chooses the TCA representation, then the static constant from the TCA model would become an input to the glycolysis model, effectively clamping pyruvate concentration in the merged system. If the user chooses the glycolysis representation, then the glycolysis variable becomes an input to the TCA cycle model, and any variables that previously depended on the static constant become mathematically dependent on the output of the glycolysis model instead. For more detailed examples of other Type 3 compositions using SemGen, see Gennari et al. [14], Beard et al. [13], and Neal [15].

Type 3 model composition offers the traditional benefits of a modular modeling architecture in that modelers can construct systems from externally developed, interoperable pieces, but the component models do not have predefined interfaces. Thus, the components used in these types of compositions do not meet the definition of a module put forth in the Introduction. We therefore reify this new approach to modular model composition, naming it SAIM. To our knowledge, this compositional approach has not been explicitly defined previously. This may be because it requires two key technological ingredients that have appeared only recently: model interchange formats for community-level reuse and semantic web standards that can provide machine-readable definitions of model components.

Although they do not have predefined interfaces, SAIM components retain an independent identity apart from the systems in which they are used (each component used in an integration task originally exists apart from the merged system). It is only when these components are incorporated into a larger system that the input/output connections between them are established. This way, SAIM avoids the “inaccessible variable problem” associated with traditional, information-hiding approaches for software composition articulated in the Introduction. While Type 2 compositions also avoid these problems by applying a white-box approach, SAIM provides an important advantage that can expedite and simplify the model composition process: in lieu of predefined interfaces, the model's semantics provide a guide for how to couple modeling components. Interfaces are one of the hallmarks of traditional modular designs because they make component coupling simpler and more intuitive. If they are removed in favor of white-boxing the components' contents, system creators must have a detailed understanding of each component's internal structure before they can appropriately link them. This, in effect, reduces much of the timesaving compositional power that a modular design is supposed to provide. Therefore, effectively guiding component coupling is one of the primary challenges for any white-box compositional approach. To provide a more concrete example, consider that linking household audio components does not require the user to understand their internal circuitry, only where to connect the cables. In the absence of predefined interfaces on these components, one would need circuit-level knowledge of their internal structure to link them appropriately. In biological modeling, SAIM helps retain some of the compositional power that is lost by eliminating predefined interfaces: the machine-readable biological annotations on SAIM components can be used to propose inter-model links at the time of merging. This allows modelers to connect modeling components in terms of their biology, not necessarily their code. SemGen and semanticSBML provide this semantics-guided approach: both use metadata annotations from a core set of reference ontologies to identify the biological commonalities between models, suggest the appropriate links to merge them, and then make the necessary changes to equations, physical units, etc., when the merged model is exported for simulation (Figure 1B).

## Discussion

Generally speaking, our intention is not to advocate any specific type of model composition over another. Each approach described here has advantages and disadvantages. Our intention is to point out the specific advantages that SAIM provides within the context of community-wide modular modeling and reuse. The standard approach to modularity used in software engineering is problematic in this context because information-hiding interfaces can limit the ways modeling components can be recombined. SAIM provides a solution by white-boxing model contents and then guiding the coupling process using machine-readable descriptions of the model's underlying biology. After this process, the model could once again be used in a black-box fashion, if desired.

Also, it is not our intention to champion any particular biological modeling format. Collectively, we constitute a group of modelers who are active in the development of CellML, SBML, and the SemSim framework; therefore, our perspective on model reuse and modularity extends across these, and other, formats. CellML, SBML, and SemSim are each expressive enough to allow all three styles of model composition that we have discussed. In our view, enabling such composition is primarily a matter of developing the software technologies that support working with these models in the different compositional styles. For example, while CellML models do use predefined interfaces on modeling components, software can turn these black boxes into white boxes without altering the original CellML model (as in the Antimony package). Each block of mathematical equations in a CellML component can be analyzed to determine how each variable in the component is computed irrespective of its input/output designation. Furthermore, Resource Description Framework (RDF)-formatted semantic metadata can be applied to CellML models to support SAIM (Type 3) compositions, and the CellML community is currently testing new features in SemGen that support the semantic annotation of these models.

**Figure 2:**
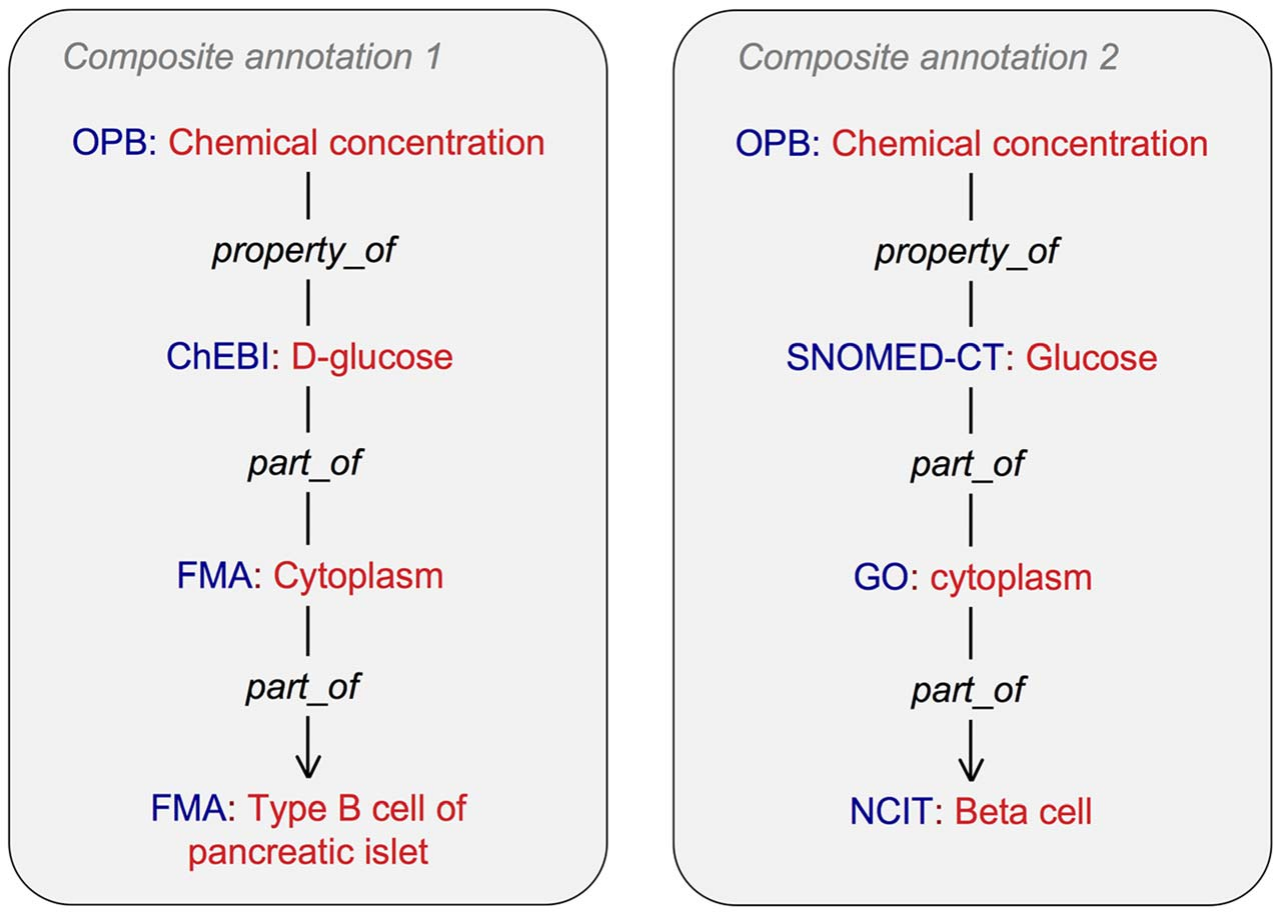
Two possible composite annotations that represent the concept “cytoplasmic glucose concentration of pancreatic beta cell.” Although the annotations are semantically equivalent, an automated model-merging tool may not recognize them as such, as their component terms originate from different ontologies. Adhering to an agreed-upon set of orthogonal ontologies may help model annotators address this challenge. Abbreviations: OPB, Ontology of Physics for Biology; ChEBI, Chemical Entities of Biological Interest; SNOMED-CT, Systematized Nomenclature of Medicine—Clinical Terms; FMA, Foundational Model of Anatomy; GO, Gene Ontology [22]; NCIT, National Cancer Institute Thesaurus [23].

To realize the advantages that semantics-based composition offers in terms of model sharing and reuse, biological content within models must be precisely and thoroughly annotated. This presents a number of challenges. First, despite the rich set of biomedical ontologies and controlled vocabularies now in existence, they often cannot provide the level of semantic detail that is required for annotating biological models precisely. For example, while “cytoplasmic glucose concentration of pancreatic beta cell” is simulated in several publicly available models of pancreatic beta cell dynamics, to our knowledge it is not a concept that is currently represented in any biomedical ontology or other knowledge resource. To address this limitation, the developers of SemSim and SemGen have devised a composite annotation approach [14] in which existing terms in biomedical knowledge resources are linked via standardized ontological relations to form the new, more exact terms needed for annotating concepts in biological models. Using this approach, a composite annotation for “cytoplasmic glucose concentration of pancreatic beta cell” can be created by linking “Chemical concentration” from the Ontology of Physics for Biology [17], “D-Glucose” from Chemical Entities of Biological Interest (ChEBI), “Cytoplasm,” from the Foundational Model of Anatomy (FMA) [18], and “Type B cell of pancreatic islet” from the FMA (Figure 2, left).

Another challenge associated with model annotation is that annotations must be consistent for the same biophysical content across models. Otherwise, recognizing the points of semantic overlap during model merging becomes difficult. Controlled biomedical knowledge resources such as ontologies can provide a standard set of reference terms to achieve this consistency, and we advocate their use in the annotation process; however, these knowledge resources can contain significant semantic overlap, which can result in inconsistencies between annotators. Figure 2 demonstrates that an alternative, semantically equivalent composite annotation for “cytoplasmic glucose concentration of pancreatic beta cell” could be created using terms from a different set of ontologies. For example, while one annotator may refer to the concept “D-glucose” from the ChEBI knowledge base [19], another may refer to the term “Glucose” from the Systematized Nomenclature of Medicine—Clinical Terms resource (SNOMED-CT [20]). We believe a tenable solution to this problem is for the various active model curation groups to agree on a core set of orthogonal reference ontologies for use in biological annotation (see, for example, the efforts to develop the CellML Biological Annotation Metadata Specification—https://cellml-metadata-spec-20.readthedocs.org/en/latest/cellmlmetaspec-biological.html). Our hope is that this solution will promote consistency among annotators within and between model curation groups. We also encourage a sustained, community-wide discussion on semantic annotation standards across modeling formats and domains. It is important that this discussion involve members of both the modeling and knowledge representation communities, not only to ensure consistency in annotation efforts but because as new scientific knowledge is articulated in mathematical models, it will be crucial that knowledge resource developers incorporate this information into existing resources so that model annotators have timely access to emerging biological concepts. Furthermore, as scientific knowledge evolves, terms previously used for annotation may become obsolete. Therefore, a dynamic collaboration between modelers and knowledge resource developers is also important for ensuring that such annotations can be replaced with terms representing more current scientific understanding.

We recognize that creating unambiguous annotations can be time-consuming and requires expertise in existing ontologies. Thus, critical challenges for SAIM modeling include incentivizing model curators to annotate at this level of detail and developing intuitive software that minimizes annotation costs. In terms of incentives, annotation provides important advantages for model sharing, modularity, and reuse. It also provides a basis for linking models with other annotated knowledge resources, including experimental datasets. For modelers, such links can help uncover datasets useful for driving and/or validating models of interest. For experimentalists, they can help uncover biological models that show how the elements of a biological system under investigation influence each other. As demonstrated recently, semantic annotations can also be used to organize the biological content of model collections into integrated knowledge resources that can represent causal biological pathways across multiple physical and temporal scales [21]. A rich semantic layer applied to models can also greatly facilitate the search and retrieval of models and their sub-components within online repositories [10], [21].

As more and more biological models become publicly available, researchers will have more opportunities to build upon previous modeling efforts and investigate complex, integrated biological systems in silico. By taking advantage of this growing number of sharable models, we anticipate that modular modeling solutions will significantly accelerate quantitative research in systems biology, synthetic biology, integrated physiology, and all other fields that utilize complex biological models. We hope that our arguments presented here will inform the development of these solutions and ensure their utility across the greater modeling community.
